# Systemic and Ocular Hemodynamic Risk Factors in Glaucoma

**DOI:** 10.1155/2015/141905

**Published:** 2015-10-18

**Authors:** Jaewan Choi, Michael S. Kook

**Affiliations:** ^1^Central Seoul Eye Center, 224 Ichon-ro, Yongsan-gu, Seoul 04427, Republic of Korea; ^2^Department of Ophthalmology, University of Ulsan, Asan Medical Center, Seoul 05505, Republic of Korea

## Abstract

Primary open angle glaucoma (POAG) is a multifactorial disease characterized by progressive retinal ganglion cell death and visual field loss. It is known that alterations in intraocular pressure (IOP), blood pressure (BP), and ocular perfusion pressure (OPP) can play a significant role in the pathogenesis of the disease. Impaired autoregulatory capacity of ocular blood vessels may render tissues vulnerable to OPP changes and potentially harmful tissue ischemia-reperfusion damage. Vascular risk factors should be considered more important in a subgroup of patients with POAG, and especially in patients with normal tension glaucoma (NTG) with evidence of unphysiological BP response. For example, reduction of BP during the nighttime has an influence on OPP, and increased circadian OPP fluctuation, which might stand for unstable ocular blood flow, has been found to be the consistent risk factor for NTG development and progression. Central visual field may be affected more severely than peripheral visual field in NTG patients with higher 24-hour fluctuation of OPP. This review will discuss the current understanding of allegedly major systemic and ocular hemodynamic risk factors for glaucoma including systemic hypertension, arterial stiffness, antihypertensive medication, exaggerated nocturnal hypotension, OPP, and autonomic dysregulation.

## 1. Introduction

Glaucoma is the leading cause of irreversible blindness worldwide, affecting more than 60 million people worldwide [1]. The disease is characterized by progressive loss of retinal ganglion cells and their axons associated with tissue remodeling in the optic nerve head (ONH). Corresponding visual field (VF) deterioration may progress in the area of anatomical ONH and retinal nerve fiber layer (RNFL) damage if the disease is not controlled. Glaucoma is a multifactorial disease, but its precise pathogenesis remains still unclear. Although intraocular pressure (IOP) is the most important risk factor in the development and progression of glaucoma, reducing the IOP does not guarantee the cessation of the disease progression [2–6]. Some patients show glaucoma progression despite the low IOP maintenance [6].

Primary open angle glaucoma (POAG) and normal tension glaucoma (NTG) share similar risk factors for the pathogenesis, which can be largely classified under mechanical and vascular categories. Mechanical risk factors include increased IOP, thinner lamina cribrosa (LC), larger LC displacement, anatomical variations of LC, or translaminar pressure dynamics [7–10].

However, alteration of systemic blood pressure (BP) or ocular blood flow (OBF) also seems to be involved in the disease process [11]. Several studies have shown that alterations in systemic and ocular hemodynamics play a significant role in the development and progression of glaucoma [12–15]. These vascular risk factors can be largely classified under some categorizations such as BP-related components, OPP-related components, autonomic dysregulation, or other pathologic vascular conditions like arteriosclerosis that may compromise OBF.

The understanding of the relationship between these risk factors and glaucoma has not been established well. For example, systemic high BP may increase OPP theoretically, as BP is a major mathematical contributor in calculating OPP. However, it is inappropriate to regard high OPP which resulted from systemic hypertension as decreasing the risk of POAG and NTG for all age groups. Based on the current studies, systemic hypertension has different effect on the development of POAG in different age groups.

This review article will summarize the present knowledge on several systemic and ocular hemodynamic risk factors in the pathogenesis of POAG and NTG and try to discuss the relationship among risk factors. The detailed topics will include systemic hypertension, arterial stiffness, antihypertensive medication, exaggerated nocturnal hypotension, mean ocular perfusion pressure (MOPP), and autonomic dysregulation. In the latter part of the paper, vascular pathogenesis theory based on the role of unstable OPP and autonomic dysregulation of the vessels will be highlighted.

## 2. Systemic Hypertension

Systemic hypertension causes target organ damage (TOD) that involves vasculature, heart, brain, and kidneys. Complex biochemical, hormonal and hemodynamic mechanisms are involved in the pathogenesis of TOD. Common to all these processes is an increased bioavailability of reactive oxygen species (ROS) [16].

It is well known that systemic hypertension accelerates atherosclerotic change that induces structural modifications of the arterial wall and reduction of vessel wall compliance. At its initial stage of hypertension, the blood flow may increase as BP increases. However, as irreversible vessel wall damage and endothelial hypertrophy progress, blood flow finally decreases. Hypertension more significantly compromises the vessel function in older people. For example, older subjects with hypertension are at much higher the risk of cardiovascular complications [17] and have narrow range of autoregulation [18].

However, the role of systemic hypertension in the change of the optic nerve system related to glaucoma has not been elucidated very clearly. In meta-analyses, systemic hypertension increases the risk of developing POAG [19, 20]. The association between systemic hypertension and POAG was stronger in cross-sectional compared with case-control and longitudinal studies, supporting a role of increased BP in the elevation of IOP, and possibly in the development of glaucoma [19]. Systolic and diastolic BP showed modest, positive association with POAG. The effect of BP on POAG was modified by age, with a stronger association among older subjects. Nearly 8% of the adults with systemic hypertension had glaucoma with the highest prevalence of NTG [21]. Systemic hypertension may contribute to increased IOP via overproduction or impaired outflow of aqueous humor [22, 23]. Some population-based studies have found statistically significant positive associations of systolic and diastolic BP with IOP [24–28], whereas others did not [29–31]. The Baltimore Eye Survey showed that systemic hypertension had protective effect on POAG in subjects with age less than 60 years, and adverse effect on those with age more than 70 years [32]. This finding may be explained by the assumption that younger people with no vessel damage yet may take advantage of high BP for increasing OPP, while the older people with narrowed vessel lumen may have decreased OPP and subsequent glaucomatous damage to the ocular structures.

## 3. Arterial Stiffness

Arterial stiffness, a loss of arterial elasticity, is one of the major signs of vascular aging [33]. Increased arterial stiffness has been recognized as an independent risk factor for cardiovascular diseases [34]. Some researchers have investigated the association between arterial stiffness and glaucoma. However, the role of arterial stiffness in the pathogenesis of glaucoma is still controversial. The parameters that may have association with arterial stiffness include central BP, arterial waveform analysis derived from pulse tonometry, or dynamic retinal vessel reactivity analysis to flicker light [35–38].

Brachial BP or derived central aortic BP parameters generally showed no significant differences between glaucoma patients and normal individuals. The POAG group had a lower pulse pressure, which was also significant in the regression analysis (*P* < 0.002). In the arterial waveform analysis, POAG was associated with a lower brachial form factor than NTG (*P* < 0.001) and higher subendocardial viability ratio (*P* < 0.008) [35]. Another cross-sectional study in which POAG and age- and sex-matched controls were compared showed that there was no difference in heart rate and pulse pressure between patients with glaucoma and the control subjects [36]. However, both distensibility coefficient of common carotid artery and baroreflex sensitivity were reduced in POAG patients, and stiffness (characterizing the rigidity of vessel walls) was higher in the POAG patients than in the controls using the ultrasound wall tracking system.

Similar relationships suggesting a pathological large artery function including decreased baroreflex sensitivity were found in pseudoexfoliation glaucoma with the same method [37]. Another study also showed that there is a positive correlation between the presence of pseudoexfoliation glaucoma and carotid-femoral pulse wave velocity [39].

In a study in which dynamic retinal vessel reactivity analysis to flicker light was performed, both POAG and NTG groups exhibited steeper retina arterial constriction slopes after cessation of flicker (*P* = 0.007), and a similarly increased fluctuation in arterial and venous baseline diameter (*P* = 0.008 and *P* = 0.009, resp.) compared with controls [38].

According to the Rotterdam Eye Study, participants with an increased pulse wave velocity and especially those with a low carotid distensibility coefficient, both indicative of high arterial stiffness, had a higher prevalence of POAG, although results were not statistically significant. No associations were found between parameters of arterial stiffness and NTG [40]. Another report also showed that there was no difference in brachial-ankle pulse wave velocity among POAG patients, NTG patients, and controls, when arterial stiffness was interpreted by the parameter of brachial-ankle pulse wave velocity [41].

The inconsistent results between studies may be due to the unstandardized research modalities to assess the arterial stiffness and the different chronic open-glaucoma subgroups including POAG, NTG, and PXFG. For example, PXFG is a subgroup of chronic open-angle glaucoma but has a different aspect with the deposition of extracellular fibrillar material on the anterior segment of eyeball including the trabecular meshwork from POAG and NTG [39, 42, 43]. The pseudoexfoliation fibers are consistently associated with connective-tissue components throughout the body, particularly fibroblasts and collagen and elastic fibers, myocardial tissue specimens, and heart-muscle cells, thus possibly being associated with arterial stiffness [36, 39]. From the previous studies, we think that arterial stiffness may be associated with the pathogenesis of open-angle glaucoma, but not with all subgroups.

## 4. Antihypertensive Treatment

There have been ongoing debates concerning the effect of systemic antihypertensive treatment on the incidence of POAG. Because systemic hypertension generally is treated with oral antihypertensive medication, it is needed to distinguish the effect of systemic antihypertensive medication from systemic hypertension itself. Systemically, the use of antihypertensive drugs is considered to be one of the major factors contributing to the prevalence of postural hypotension [44, 45], although other studies have failed to find a significant causal association [46, 47]. The relationships between orthostatic hypotension and circadian BP variability have been investigated by many researchers [48–50].

There are some evidences showing that the structural changes may be induced from taking systemic antihypertensive medication. Thessaloniki Eye Study revealed that diastolic BP less than 90 mmHg that resulted from antihypertensive treatment was associated with increased cupping and decreased rim area of the optic disk measured by the Heidelberg Retinal Tomography parameters in nonglaucomatous subjects [51, 52]. This association was not found in the untreated group with diastolic BP <90 mmHg, nor in the group with diastolic BP >90 mmHg using antihypertensive treatment. Similar association between optic disc image analysis and low PP was also found in subjects treated for systemic hypertension by subgroup analysis [51].

A subsequent study found out that angiotensin-converting enzyme inhibitors/angiotensin receptor blockers, diuretics alone or combined with other medications, and other combinations of antihypertensive treatment were significantly associated with larger cup size and higher C/D ratio compared with the untreated group [53]. However, this association was not found for subjects receiving beta-blocker and/or calcium channel blocker. When the analysis was repeated following stratification by BP level, all classes of antihypertensive medications were significantly associated with larger cup size and higher C/D ratio in subjects with DBP <90 mmHg treated for systemic hypertension, compared with untreated subjects with the same BP level. This suggests that the associations are mediated through the lowered BP per se and not the specific mechanism of action of the antihypertensive medications.

Rotterdam Eye Study also revealed that lower diastolic perfusion pressure in persons taking antihypertensive medication was associated with the higher prevalence of high tension OAG. The odds ratio for high tension OAG was 4.68 for subjects with diastolic PP <50 mmHg when compared with those with DPP >65 mmHg in persons taking antihypertensive medication [40].

In cardiology, it has been known that antihypertensive treatment may cause coronary artery perfusion pressure to fall below the low threshold in patients with coronary stenosis and to aggregate myocardial ischemia [54]. This speculation may be extended to the pathogenesis of glaucoma in certain types. Antihypertensive medication may cause nonphysiologic hypotension, rendering systemic pulse pressure and OPP fluctuation wider in subjects with autonomic dysfunction, and finally causing ischemia-reperfusion damage to the optic disc in glaucoma pathogenesis.

## 5. Exaggerated Nocturnal BP Reduction

Normal healthy people have physiological nocturnal BP reduction of about 5 to 10% at nighttime when compared with that of daytime. The remaining individuals are classified as either nondippers or overdippers [55, 56]. Physiologic BP reduction at nighttime is caused by a reduction in sympathetic activity during the night with a reduced amount of circulating catecholamine hormones, which can in turn lead to a decrease in heart rate, cardiac input, and peripheral resistance. This response may be augmented or blunted in certain subsets of population with vasospastic disorders, orthostatic hypotension, atherosclerosis, or obstructive sleep apnea syndrome. The use of corticosteroid or systemic antihypertensive medication may be associated with this phenomenon [50, 57–63].

24-hour ambulatory BP monitoring can be performed to reveal the characteristics of circadian BP variability in subjects with glaucoma [64]. Many studies have found out that exaggerated nocturnal BP fall is a risk factor for progressive VF loss in glaucoma [65–70]. The odds ratio for deteriorating VFs over 2 years with nocturnal dips >10% in systolic or diastolic BP was 3.32 (1.84–6.00) and 2.09 (1.20–3.64), respectively [65]. In patients with glaucoma and well-controlled hypertension, a nocturnal BP fall of more than 10% is associated with both advanced functional VF deficits and greater structural damages of ONH [66]. NTG eyes with VF deterioration had significantly (*P* = 0.05) lower minimum nighttime diastolic BP, and the use of oral hypotensive therapy on systemic hypertension was associated with a significantly lower mean nighttime systolic BP (*P* = 0.006) [67]. In this study, larger mean percentage drop in systolic (*P* < 0.0001), diastolic (*P* = 0.0009), and mean (*P* < 0.0001) BPs were associated with VF progression in NTG eyes. Patients with progressive glaucoma tend to have lower BP at nighttime than stable glaucoma patients. Patients who had VF progression showed significantly lower nocturnal BP variables, with the dips of the systolic, diastolic, and mean arterial pressure (MAP) significantly larger [69]. In a prospective longitudinal cohort study, cumulative nocturnal hypotension predicted VF loss. They showed that the total time that MAP during sleep was 10 mmHg below the daytime MAP was a significant predictor of global VF progression by multivariate analysis (*P* < 0.02) [70].

There are some studies showing an increased tendency of VF progression in both the nondipper and the extreme dipper group than in the physiologic dipper group [68]. Systemically, nondippers have a high incidence of cardiac and vascular diseases, whereas extreme dippers show greater progression of asymptomatic cerebrovascular disease than physiologic dippers.

**Figure 1:**
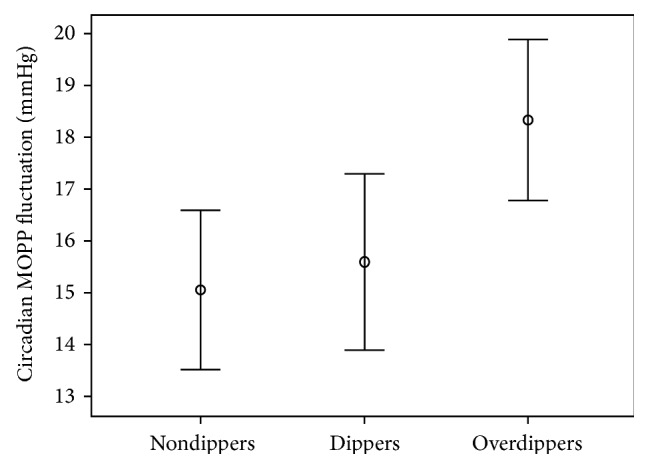
Circadian mean ocular perfusion pressure (MOPP) fluctuation (CMF) in patients with normal tension glaucoma (NTG), classified by different blood pressure pattern (nondippers, dippers, and overdippers). Overdippers had a significantly larger CMF than did the other groups on post hoc comparison test (Dunnett's test; *P* = 0.007 between nondippers and overdippers, and *P* = 0.035 between dippers and overdippers). Error bars, 95% confidence interval (adapted from [72]).

The mechanism of how exaggerated nocturnal BP reduction affects the development and progression of glaucoma is not clearly known yet. Yazici et al. found that excessive and repetitive nocturnal BP decreases occur more frequently in some patients with NTG, compared with those with POAG or ocular hypertension [71]. Choi et al. suggested a hypothesis that nocturnal BP reduction affects the circadian variability of ocular blood flow expressed in the term of MOPP. They showed that fifty-five (41.7%) of 132 patients with NTGs were classified into the overdipper group, and marked circadian MOPP fluctuation was noted in this group when compared with the nondippers and physiologic dippers (Figure 1) [72]. Interestingly, circadian MOPP fluctuation showed positive associations with VF indices at initial diagnosis of NTG, suggesting that daily repetitive ischemic insults to ocular structures may be an underlying mechanism of glaucomatous optic neuropathy.

## 6. Mean Ocular Perfusion Pressure

There have been numerous efforts to elucidate the relationship between OBF and the pathogenesis of POAG and NTG. The modalities for OBF measurement include color Doppler imaging of ophthalmic artery and short posterior ciliary artery [74], laser Doppler flowmetry [75, 76], laser speckle flowmetry [77, 78], pulsatile ocular blood flow analyzer [79], and laser interferometric measurement of fundus pulsation [80]. Lower perfusion pressure (PP, BP-IOP) was strongly associated with an increased prevalence of POAG, with a sixfold excess for those in the lowest category of PP [32], suggesting that POAG is associated with an alteration in factors related to OBF and a breakdown of autoregulation. These findings were echoed in the Rotterdam Eye Study [40], the Barbados Eye Study [81], and the Egna-Neumarkt Study [23].

The amount of blood influx to the ocular structures may be also estimated through the parameter of MOPP. MOPP is the parameter derived from the difference of mean arterial pressure (MAP) and IOP. MAP and MOPP are calculated from systolic blood pressure (SBP), diastolic blood pressure (DBP), and IOP as follows: (1) MAP = DBP + [1/3 × (SBP − DBP)], (2) MOPP = 2/3 × MAP – IOP, and (3) circadian MOPP fluctuation = peak MOPP − trough MOPP. Unstable OBF may be a risk factor for open-angle glaucoma. BP, IOP, or vascular dysregulation may render the ocular perfusion pressure unstable and cause ischemia-reperfusion damage to the ganglion cells by oxygen free radicals and nitric oxide species. Diurnal variability of MOPP was associated with the development of POAG. Sehi et al. demonstrated that relative diurnal change in IOP did not differ between patients with untreated POAG and age-matched normal subjects. They also found that the percentage decrease in diurnal MOPP was significantly larger in patients with untreated POAG than in normal subjects, suggesting that relative diurnal change in MOPP may be a risk factor for POAG [82].

Choi et al. found that marked circadian MOPP fluctuation was associated with nocturnal BP reduction. In the series of 132 NTG patients who had 24-hour ambulatory BP and IOP monitoring, they found out that MOPP significantly decreases at night and circadian MOPP fluctuation increases in BP over-dipper group. Increased circadian MOPP fluctuation was proportional to advanced structural and functional damage measured by Humphrey VF perimetry and scanning laser polarimetry in NTG patients at initial evaluation, implying the parameter as a possible risk factor for the development of NTG [72]. Their subsequent study evaluated the strength of the circadian MOPP fluctuation as a risk factor for NTG development. Using multivariate regression analysis, the circadian MOPP fluctuation was the most frequent predictor for advanced structural and functional glaucomatous damage [83].

Circadian MOPP fluctuation seems to be related with VF progression in NTG eyes. In cohort studies using Kaplan-Meyer survival analysis, the group with highest circadian MOPP fluctuation showed a greater cumulative probability of central VF progression within 10 degrees than the group with lowest circadian MOPP fluctuation, by both linear regression analysis and modified Anderson criteria [84, 85]. In multivariate analysis, only circadian MOPP fluctuation was significantly associated with central VF progression [84]. Peripheral VF progression was not different among groups classified by the degree of circadian MOPP fluctuation. The risk factors for central VF defect progression were increased circadian MOPP fluctuation and worse baseline mean deviation value at initial visual field test [85].

**Figure 2:**
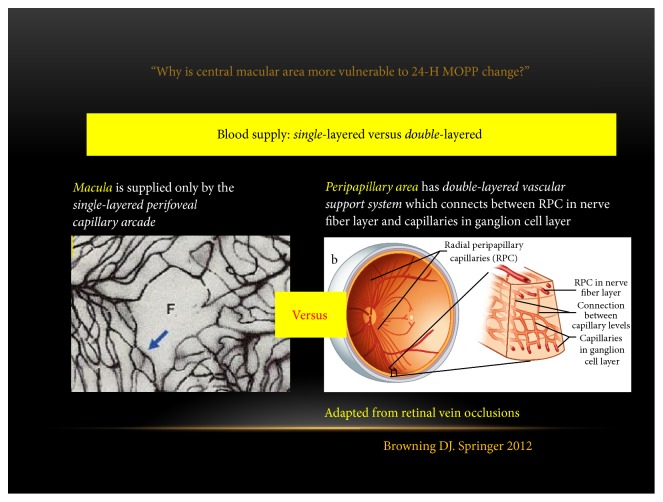
Different vascular support system between macula and peripapillary area. (1) Macula is only supplied only by the single-layered perifoveal capillary arcade. (2) Peripapillary area had double-layered vascular support system which connects between radial peripapillary capillaries (RPC) in retinal nerve fiber layer and capillaries in ganglion cell layer. MOPP, mean ocular perfusion pressure (adapted from [73]).

The possible reason why central macular area seems more vulnerable to circadian MOPP fluctuation may be the different vascular support system between macular and peripapillary area. Macula is supplied only by the single-layered perifoveal capillary arcade. In contrast, peripapillary area has double-layered capillary system which consists of radial peripapillary capillaries in retinal nerve fiber layer and capillaries in ganglion cell layer [73]. Thus, we can speculate that if ischemia-reperfusion damage happens repeatedly, the macular area may be more easily damaged than the peripapillary area (Figure 2).

## 7. Primary Vascular Dysregulation

Vascular dysregulation refers to the regulation of blood flow that is not adapted to the needs of the respective tissue. In normal healthy eyes, the retinal blood flow is autoregulated and is independent of OPP within a certain range [86]. However, the autoregulation of OBF may be disturbed in glaucomatous eyes, and the physiologic response may be different from that in normal healthy eyes. When scanning laser Doppler flowmetry was performed to measure blood flow in the temporal neuroretinal rim and the cup of the optic nerve head (ONH) and pulsatile choroidal blood flow was assessed by laser interferometric measurement of fundus pulsation amplitude, a significant positive correlation between laser Doppler flowmetry readings and MAP was observed in patients with glaucoma but not in healthy control subjects. Likewise, the correlation coefficient between fundus pulsation amplitude and MAP was higher in patients with glaucoma than in healthy control subjects [87]. This study implies that proper autonomic regulatory response may be missing in glaucoma. Harris et al. also demonstrated the presence of a reversible vasospasm specifically within the ocular vasculature of patients with NTG compared with the normal control, using color Doppler imaging and hypercapnia [88].

Insufficient or improper adaption of BF, despite anatomically healthy vessels and the absence of a causative disease, is termed* primary vascular dysregulation*. In subjects with PVD, retinal vessels are stiffer and more irregular, and both neurovascular coupling and autoregulation capacity are reduced while retinal venous pressure is often increased [89]. The combination of PVD with a cluster of additional vascular and nonvascular signs and symptoms is called PVD syndrome or Flammer syndrome [90]. Despite many symptoms and signs, most subjects with Flammer syndrome are healthy subjects. However, subjects with Flammer syndrome are at high risk of developing NTG [91]. The OBF instability in these subjects with limited range of autoregulation seems to predispose the optic disc structures at risk of ischemia-reperfusion damage, finally leading to the development and progression of open-angle glaucoma [92].

## 8. Concluding Remarks

**Figure 3:**
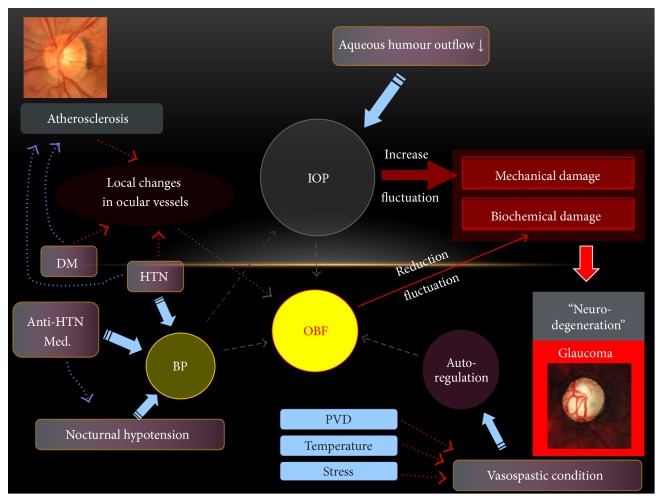
Schematic representation on the complex relationships among various glaucoma risk factors. BP, blood pressure; IOP, intraocular pressure; OBF, ocular blood flow; HTN, hypertension; PVD, primary vascular dysregulation.

Various risk factors have been investigated in systemic and ocular hemodynamic risk factors for glaucoma. Every risk factor is complexly linked to each other. IOP-related mechanical stress, BP-related vascular stress, and disturbed autoregulation are complexly linked to each other in the development and progression of open-angle glaucoma (Figure 3). Daily repetitive ischemia-reperfusion insults to ocular structures may play a role in the vascular pathogenesis of NTG. Vascular etiology should be considered as more important, especially in the NTG eyes with systemic and ocular hemodynamic risk factors, and in the eyes showing central VF defect progression. Although the underlying relationship between risk factors seems unclear yet and the evidence-based treatment is still lacking based on vascular etiology, the clinicians and researchers always should be aware of the vascular origin of glaucoma pathogenesis.
